# Prevalence of Undernutrition and Its Associated Factors Among Pregnant Women Attending a Healthcare Facility in Kabul, Afghanistan

**DOI:** 10.1002/puh2.70453

**Published:** 2026-11-07

**Authors:** Zainab Ezadi, Maliqa Mohaqiq, Agus Fitriangga, Muhammad Haroon Stanikzai, Robaba Edalat, Massoma Jafari

**Affiliations:** ^1^ Reproductive Health Cure Hospital Kabul Afghanistan; ^2^ Health Net TPO Kabul Afghanistan; ^3^ Department of Community Medicine Faculty of Medicine Tanjungpura University Pontianak Indonesia; ^4^ Department of Public Health Faculty of Medicine Kandahar University Kandahar Afghanistan; ^5^ Agency for Assistance and Development of Afghanistan Kabul Afghanistan; ^6^ Department of Health Professions Education Research University of Toronto Toronto Ontario Canada

**Keywords:** Afghanistan, associated factors, Kabul, pregnant women, undernutrition

## Abstract

**Background:**

Maternal undernutrition remains a major public health challenge in low‐resource settings, with significant implications for maternal and child morbidity and mortality. There is limited evidence on the factors associated with undernutrition among pregnant women in Afghanistan. Therefore, this study aims to assess the prevalence of undernutrition and its associated factors among pregnant women attending a healthcare facility in Kabul, Afghanistan.

**Methods:**

We conducted a cross‐sectional study from April 1 to August 7, 2025, among 421 pregnant women attending Pol‐e‐Khoshk Comprehensive Healthcare Center (CHC) in Kabul, Afghanistan. Data were collected using systematic random sampling. Undernutrition was defined as a mid‐upper arm circumference (MUAC) <23 cm. Bivariate and multivariable logistic regression analyses were performed to identify factors independently associated with undernutrition. Statistical significance was declared at *p* value <0.05, and adjusted odds ratios (AORs) with 95% confidence intervals (CIs) were reported.

**Results:**

Of the 421 pregnant women, 52% (95% CI: 47.13%–56.88%) were undernourished. In the adjusted model, the odds of undernutrition were greater in women with increasing age (AOR = 1.17; 95% CI: 1.02–1.27), those with difficulty in accessing food (AOR = 2.78; 95% CI: 1.73–4.47), and those consuming less than three meals per day (AOR = 2.78; 95% CI: 1.14–6.70).

**Conclusion:**

About half of Afghan women in this study had undernutrition. Maternal age, difficulty in accessing food, and consuming less than three meals per day were significantly associated with undernutrition. Therefore, targeted interventions addressing the factors identified in this study are worth considering.

## Graphical abstract

A facility‐based cross‐sectional study among 421 pregnant women found a 52.0% prevalence of undernutrition (MUAC <23 cm). Undernutrition was significantly associated with older maternal age, difficulty accessing food, and reporting less than three meals per day, highlighting the need for context‐specific maternal nutrition interventions.

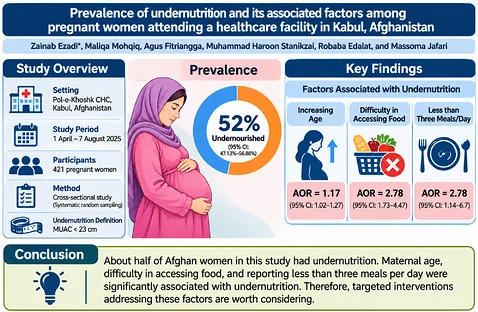

## 1 Background

Undernutrition, defined as insufficient intake of energy and nutrients to maintain good health, remains a global public health problem, directly or indirectly contributing to the deaths of 3.5 million women annually, mainly in low‐ and middle‐income countries (LMICs) [1, 2, 3]. Worldwide, 6.9 million pregnant women (25%) are undernourished, particularly in countries experiencing severe crises [4]. Maternal undernutrition is strongly associated with adverse pregnancy outcomes, including low birth weight, stunting, and increased maternal and fetal morbidity and mortality [5].

These early nutritional deficits during the first 1000 days of life have significant, lasting effects that can impact an individual for their entire life [6]. Mothers who experienced undernourishment during their own childhoods are at a higher risk of giving birth to babies with low birth weight, thereby continuing the cycle of undernutrition across generations [7]. Despite global commitments, maternal undernutrition persists, affecting nearly 10% of women worldwide [8].

At the regional level, studies highlight significant variation in prevalence and risk factors. A study in northern Ethiopia reported a prevalence of 40.6%, with key risk factors, including maternal occupation, malaria, undesired pregnancy, coffee intake, and anemia [9]. Similarly, a study in southern Ethiopia documented a prevalence of 21.8%, with risk factors such as maternal age, short birth intervals, lack of dietary modification during pregnancy, and poor nutrition knowledge [10].

Several countries in South Asia face a dual challenge of undernutrition, characterized by the presence of both undernutrition and overnutrition, along with a significant increase in noncommunicable diseases [11, 12, 13]. In Pakistan, the prevalence of undernutrition among pregnant and lactating women remains alarmingly high, with nearly 46% affected when measured by mid‐upper arm circumference (MUAC) <21 cm [14].

In Afghanistan, the situation is particularly dire [15]. Anemia affects one in three teenage girls [16], and a study in Helmand province showed that the prevalence of anemia was 51% among pregnant women [17]. The United Nations International Children's Emergency Fund (UNICEF) estimated that 28% of Afghan women marry before the age of 18, making them more vulnerable to complications in pregnancy and childbirth [18]. Poverty, illiteracy, political instability, poor diet quality, limited food access, and high reproductive frequency create significant barriers to optimal nutrition for Afghan women [6, 19]. The political changes in August 2021 further worsened the situation [20], rendering food unaffordable for many and leading to widespread food insecurity affecting approximately 20 million people [21]. The severity of Afghanistan's undernutrition crisis is underscored by the projection that 4 million people would suffer from acute undernutrition in 2023 [22], including 1.2 million pregnant and lactating women [23]. Micronutrient deficiencies exacerbate maternal risks, with anemia increasing hemorrhage risk [24, 25], and calcium deficiency contributing to hypertensive disorders. These deficiencies perpetuate intergenerational undernutrition and hinder maternal postpartum recovery [26, 27].

Recognizing these challenges, the World Health Organization (WHO) has set ambitious targets for 2025, including a 50% reduction in anemia and a 30% reduction in low birth weight [28]. Against this background, this study aims to determine the prevalence of undernutrition and associated factors among pregnant women in a comprehensive healthcare center (CHC) in Kabul city, Afghanistan, in 2025, providing crucial data to inform targeted interventions and contribute to achieving the WHO's 2025 goals.

## 2 Methods and Materials

### 2.1 Study Design, Period, and Settings

A facility‐based cross‐sectional study was conducted at Pol‐e‐Khoshk CHC in Kabul, Afghanistan. Pol‐e‐Khoshk CHC is located in the Chehel Dukhtaran area of District 13, west of Kabul province, and provides primary healthcare services to an estimated 48,000 people. The CHC also functions as an active facility for maternal and child undernutrition treatment and screening programs, supported by organizations such as Health Net TPO and the Agency for Assistance and Development of Afghanistan (AADA). This study was conducted between April and August 2025. The study site was selected due to its large catchment area and its recognized role as a referral center for nutrition programs in Western Kabul.

### 2.2 Study Participants

The source population of this study consisted of all pregnant women who attended antenatal care (ANC) follow‐up at the Pol‐e‐Khoshk CHC during the study period.

### 2.3 Eligibility Criteria

The criteria for inclusion we applied were all pregnant women attending the ANC service at Pol‐e‐Khoshk CHC during the study period were eligible to participate. Pregnant women who were also breastfeeding were included if they met the other eligibility criteria. Women who were severely ill at the time of data collection or who did not provide informed consent were excluded. We also excluded women who revisited the nutrition department of the CHC during the study period. Serious health conditions and severe depression can significantly alter nutritional requirements and behaviors [29, 30], which may skew study results. These conditions were identified on the basis of the participants’ self‐reported information.

### 2.4 Sample Size and Sampling Technique

The sample size required for the study was computed using a single population proportion formula. Given the absence of similar studies in a similar context, as per our search effort, we used a prevalence of undernutrition among pregnant women of 50%, a *p* value of 0.05, a 5% margin of error, and a 95% confidence interval (CI) to obtain the maximum sample size. We considered a 10% non‐response rate; the initial sample size was increased from 384 to 422.

$$n=\frac{\left(Z2\cdot p\cdot \left(1-p\right)\right)}{E2}$$

A systematic random sampling technique was used to select participants. The sampling frame consisted of pregnant women attending the ANC services at Pol‐e‐Khoshk CHC during the study period. On the basis of facility records, approximately 15 eligible pregnant women were referred to or attended the facility per day. Given the planned 104‐day data collection period, the expected number of eligible women was estimated as 1560 (15 × 104). With a required sample size of 422 participants, the sampling interval was calculated as approximately 3.7 (1560/422) and was operationalized as selecting every third eligible woman. The first participant was selected randomly from among the first three eligible women, after which every third eligible woman was invited to participate until the required sample size was reached.

### 2.5 Anthropometric Measurement

The nutritional status of pregnant women was assessed using MUAC, a practical anthropometric indicator that is relatively independent of gestational age and is therefore particularly useful for nutritional assessment during pregnancy. MUAC has also been associated with maternal and fetal outcomes and is widely used in resource‐limited and humanitarian settings [31, 32, 33, 34]. An MUAC cutoff of <23 cm was used to define undernutrition. This threshold was selected because values below 22–23 cm are associated with adverse birth outcomes, with <23 cm recommended as a conservative threshold for identifying pregnant women at risk of low birth weight, particularly in African and Asian contexts [35, 36, 37]. The <23 cm threshold has also been applied in maternal nutrition studies in South Asian populations, including Pakistan [34]. Therefore, women with MUAC <23 cm were classified as undernourished, whereas those with MUAC ≥23 cm were classified as not undernourished [38].

The health extension workers who served as data collectors took MUAC measurements. The measurement involved determining the circumference of the left upper arm, in centimeters, using a non‐stretchable MUAC tape. The measurement site was located at the midpoint between the tip of the shoulder and the elbow [31, 32, 33, 34].

### 2.6 Variables of the Study

#### 2.6.1 Dependent Variable

The dependent variable was undernutrition among pregnant women, measured using MUAC <23 cm, and categorized as undernourished or normal [31, 32, 33, 34].

#### 2.6.2 Independent Variables

Independent variables were grouped into sociodemographic characteristics, reproductive, and nutritional exposure. The sociodemographic characteristics were current age, women's education, employment status, monthly household income, place of residence, family size, type of marriage, and physiological condition of the mother. The reproductive and nutritional characteristics were mothers’ age at the first pregnancy, pregnancy intention, trimester, history of abortion, birth spacing, parity, gravidity, illness during pregnancy, use of iron/folic acid, knowledge about the importance of iron/folic acid, information about the effect of undernutrition on pregnancy and child, use of salt‐iodized, nutritional counseling, access to food, difficulties in ANC follow‐up, knowledge about undernutrition status during breastfeeding, access to clean water, and number of meals per day.

Access to food was assessed through a self‐reported question in the questionnaire regarding whether the participant experienced difficulty accessing food. Responses were categorized as “Difficult” and “Not difficult” for analysis. This variable reflects participants’ reported difficulty in accessing food and was not intended to present a comprehensive assessment of household food insecurity.

### 2.7 Data Collection

Two trained data collectors who had a background in midwifery collected the data. Both data collectors had prior experience in providing nutritional services for pregnant and lactating women as well as children under 5 years of age in healthcare centers. Prior to the study, data collectors received orientation on the study objectives, ethical considerations, and standardized data collection procedures. The data collectors conducted face‐to‐face interviews using a structured questionnaire developed based on previous studies [39, 40, 41]. Anthropometric measurements followed standard guidelines. The principal investigator checked all completed questionnaires at the end of each week for completeness of dependent variables and consistency.

### 2.8 Data Storage and Data Management

Data were coded and entered into Statistical Package for the Social Sciences (SPSS) version 26. Data were stored in a password‐protected computer accessible only to the research team. We removed all personal identifiers and assigned a code to each participant to maintain confidentiality.

### 2.9 Statistical Analysis

Data were analyzed using SPSS Statistics version 26. Descriptive statistics (frequencies, percentages, and means with standard deviations) were used to summarize participants’ sociodemographic and clinical characteristics. Associations between the outcome variable (undernutrition, defined as MUAC <23 cm) and independent variables were initially assessed using bivariate logistic regression. Variables with a *p* < 0.25 in the bivariate analyses were entered into the multivariable model. In addition, variables considered theoretically or epidemiologically important potential confounders based on the study objectives and established relationships with maternal nutritional status were considered for inclusion or retention in the multivariable model irrespective of their bivariate statistical significance. A multivariable logistic regression model was fitted to estimate adjusted odds ratios (AORs) with 95% CIs. Potential multicollinearity among independent variables was assessed using the variance inflation factor (VIF), and values ranged from 1 to 2, indicating no evidence of problematic multicollinearity. Model goodness of fit was assessed using the Hosmer–Lemeshow test, and influential observations were examined using appropriate regression diagnostics. A two‐sided *p* < 0.05 was considered statistically significant.

### 2.10 Ethics Approval and Consent to Participate

The ethical approval was obtained from the Institutional Review Board of Afghanistan, Kabul, Afghanistan (A‐3‐25‐474). All participants were provided with a clear description of the study objectives during the initial contact. Written informed consent was obtained from literate pregnant women, whereas oral consent was documented for illiterate pregnant women. Participation was voluntary, and pregnant women were informed that they could withdraw from the study at any time without any consequences. Confidentiality of responses was strictly maintained throughout the research process. Additionally, the study was conducted in accordance with ethical principles outlined in the Declaration of Helsinki.

## 3 Results

### 3.1 Sociodemographic Characteristics of the Study Participants

A total of 422 women were targeted, of whom 421 were included in the final analysis. Table 1 presents the baseline characteristics of study participants. Further, 61% of them were aged 20–29 years, 49.6% of them were illiterate, and 97.1% were homemakers. Over one‐third (38.7%, *n* = 163) of participants had less than five thousand household monthly income. Most pregnant women resided in urban areas (94.5%, *n* = 398), and over one‐quarter (26.8%, *n* = 113) of the pregnant women were in familial marriages (details in Table 1).

**TABLE 1:** Baseline characteristics of the study participants.

		Undernutrition		
Characteristics	Total *n* (%) 421 (100.0%)	No *n* (%) 202 (48.0%)	Yes *n* (%) 219 (52.0%)	*p* value
Women's age (years)				
<20	45 (10.7)	15 (3.6)	30 (7.1)	0.110
20–29	257 (61)	131 (31.1)	126 (29.9)	
30–34	77 (18.3)	39 (9.3)	38 (9)	
35+	42 (10.0)	17 (4.0)	25 (5.9)	
Women's education				
No formal education	209 (49.6)	115 (27.3)	94 (22.3)	0.014
Primary	41 (9.7)	16 (3.8)	25 (5.9)	
Secondary	150 (35.6)	59 (14)	91 (21.6)	
Higher studies	21 (5.0)	12 (2.9)	9 (2.1)	
Employment status				
Employed	12 (2.9)	6 (1.4)	6 (1.4)	0.500
Homemaker	409 (97.1)	196 (46.6)	213 (50.6)	
Monthly household income (in Afghanis)				
≤5000	163 (38.7)	78 (18.5)	85 (20.2)	0.360
5001–10,000	95 (22.6)	41 (9.7)	54 (12.8)	
10,001–20,000	14 (3.3)	5 (1.2)	9 (2.1)	
Not reported	149 (35.4)	78 (18.5)	71 (16.9)	
Place of residence				
Urban	398 (94.5)	191 (45.4)	207 (49.2)	0.520
Rural	23 (5.5)	11 (2.6)	12 (2.9)	
Family size				
≤4	122 (29.0)	56 (13.3)	66 (15.7)	0.501
>4	299 (71.0)	146 (34.7)	153 (36.3)	
Type of marriage				
Familial marriage	113 (26.8)	56 (13.3)	57 (13.5)	0.410
Nonfamilial marriage	308 (73.2)	146 (34.7)	162 (38.5)	
Physiological condition of the mother				
Pregnant women	397 (94.3)	186 (44.2)	211 (50.1)	0.050
Pregnant women and lactating mothers	24 (5.7)	16 (3.8)	8 (1.9)	

### 3.2 Reproductive and Other Related Characteristics of the Study Participants

Regarding mothers’ age at first pregnancy, the majority (90.7%, *n* = 382) of women became pregnant before the age of 25, and less than one‐tenth of participants (9.3%, *n* = 39) experienced pregnancy between 25 and 35 years. Nearly three‐quarters (71.0%, *n* = 299) of their pregnancies were intended. Over one‐third (36.6%, *n* = 154) of women were in the first trimester, and one‐quarter (23.8%, *n* = 100) had a history of abortion. Over half of the participants had <24 months of birth spacing.

Regarding parity, half of the participants (54.2%, *n* = 228) were nulliparous or primiparous, and 26.8% of the participants were primigravid. Approximately one‐third (28.5%, *n* = 120) of the participants had an illness during pregnancy. More than 10.0% of the pregnant women did not use iron/folic acid, 37.1% of participants had no knowledge about the importance of iron/folic acid, and 57.2% of the participants had no knowledge about the effect of undernutrition on pregnancy and child growth. About two‐fifths (40.6%, *n* = 171) of the participants had difficulty in accessing food. Nearly one‐third (32.8%, *n* = 138) of the participants had difficulties in ANC follow‐up due to overcrowding at the CHC. Over 1/10 (11.2%) of the participants had less than three meals per day, and 14.3% of the participants had more than three meals per day (details in Table 2).

**TABLE 2:** Reproductive and other related characteristics.

		Undernutrition		
Characteristics	Total *n* (%) 421 (100%)	No *n* (%) 202 (48%)	Yes *n* (%) 219 (52%)	*p* value
Mothers’ age at the first pregnancy				
15–24	382 (90.7)	174 (41.3)	208 (49.4)	0.001
25–34	39 (9.3)	28 (6.7)	11 (2.6)	
Pregnancy intention				
Yes	299 (71.0)	149 (35.4)	150 (35.6)	0.230
No	122 (29.0)	53 (12.6)	69 (16.4)	
Trimester of pregnancy				
1–14 weeks	154 (36.6)	72 (17.1)	82 (19.5)	0.310
15–28 weeks	164 (39.0)	74 (17.6)	90 (21.4)	
29–43 weeks	103 (24.5)	56 (13.3)	47 (11.2)	
History of abortion				
Yes	100 (23.8)	55 (13.1)	45 (10.7)	0.060
No	321 (76.2)	147 (34.9)	174 (41.3)	
Birth spacing				
≥24 months	205 (48.7)	111 (26.4)	94 (22.3)	0.004
<24 months	216 (51.3)	91 (21.6)	125 (29.7)	
Parity				
0–1	228 (54.2)	94 (22.3)	134 (31.8)	0.002
2–4	165 (39.2)	88 (20.9)	77 (18.3)	
5+	28 (6.7)	20 (4.8)	8 (1.9)	
Gravidity				
0–1	113 (26.8)	36 (8.6)	77 (18.3)	<0.001
2–4	232 (55.1)	121 (28.7)	111 (26.4)	
5+	76 (18.1)	45 (10.7)	31 (7.4)	
Illness during pregnancy				
Yes	120 (28.5)	62 (14.7)	58 (13.8)	0.220
No	301 (71.5)	140 (33.3)	161 (38.2)	
Using iron/folic acid				
Yes	374 (88.8)	181 (43.0)	193 (45.8)	0.310
No	47 (11.2)	21 (5.0)	26 (6.2)	
Knowledge of iron/folic acid				
Yes	265 (62.9)	134 (31.8)	131 (31.1)	0.160
No	156 (37.1)	68 (16.2)	88 (20.9)	
Information about the effect of undernutrition on pregnancy and child				
Yes	180 (42.8)	95 (22.6)	85 (20.2)	0.050
No	241 (57.2)	107 (25.4)	134 (31.8)	
Using salt‐iodized				
Yes	232 (55.1)	118 (28.0)	114 (27.1)	0.100
No	189 (44.9)	84 (20.0)	105 (24.9)	
Nutritional counseling				
Yes	291 (69.1)	144 (34.2)	147 (34.9)	0.340
No	130 (30.9)	58 (13.8)	72 (17.1)	
Access to food				
Difficult	171 (40.6)	57 (13.5)	114 (27.1)	<0.001
Not difficult	250 (59.4)	145 (34.4)	105 (24.9)	
Difficulties in ANC follow‐up				
Family restrictions	39 (9.3)	12 (2.9)	27 (6.4)	0.050
Overcrowding CHC	138 (32.8)	66 (15.7)	72 (17.1)	
CHC is far away	18 (4.3)	6 (1.4)	12 (2.9)	
No problem	226 (53.7)	118 (28.0)	108 (25.7)	
Knowledge about nutritional status during breastfeeding				
Yes	209 (49.6)	107 (25.4)	102 (24.2)	0.100
No	212 (50.4)	95 (22.6)	117 (27.8)	
Access to clean water				
Yes	305 (72.4)	152 (36.1)	153 (36.3)	0.210
No	116 (27.6)	50 (11.9)	66 (15.7)	
Number of meals per day				
Less than three meals per day	47 (11.2)	15 (3.6)	32 (7.6)	0.001
Three meals per day	314 (74.6)	147 (34.9)	167 (39.7)	
More than three meals per day	60 (14.3)	40 (9.5)	20 (4.8)	

Abbreviations: ANC, antenatal care; CHC, community healthcare center; CI, confidence interval.

### 3.3 Prevalence of Undernutrition Among Study Participants

As shown in Figure 1, 52% (95% CI: 47.13%–56.88%) of the pregnant women were undernourished (Figure 1).

**FIGURE 1:**
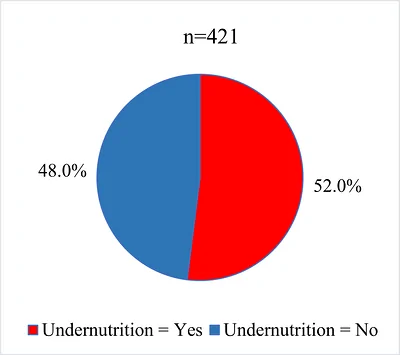
Prevalence of undernutrition among study participants.

### 3.4 Factors Associated With Undernutrition Among Pregnant Women

In multivariable logistic regression, increasing maternal age (AOR = 1.17, 95% CI: 1.02–1.27), difficulty in accessing food (AOR = 2.78; 95% CI: 1.73–4.47), and consumption of less than three meals per day (AOR = 2.78; 95% CI: 1.14–6.7) were factors associated with undernutrition among pregnant women (details in Table 3).

**TABLE 3:** Likelihood of undernutrition among pregnant women.

Characteristics	Crude odds ratio (95% CI)	*p* value	Adjusted odds ratio (95% CI)	*p* value
Maternal age (in years)	1.15 (1.06–1.24)	<0.001	**1.17 (1.02–1.27)**	**<0.001**
Women's education				
No formal education	0.91 (0.37–2.27)	0.850	1.55 (0.55–4.35)	0.390
Primary	0.48 (0.16–1.39)	0.170	0.81 (0.24–2.69)	0.730
Secondary	0.48 (0.19–1.22)	0.120	0.91 (0.33–2.51)	0.330
Higher	Reference		Reference	
Physiological condition of the mother				
Pregnant women	1.5 (0.95–2.32)	0.060	1.37 (0.84–2.23)	0.210
Pregnant women and lactating mothers	Reference		Reference	
Birth spacing				
≥24 months	Reference		Reference	
<24 months	1.62 (1.1–2.38)	0.014	1.09 (0.66–1.78)	0.721
Parity				
0–1	0.28 (0.11–0.66)	0.004	0.32 (0.11–1.19)	0.090
2–4	0.45 (0.19–1)	0.080	0.42 (0.16–1.37)	0.121
5+	Reference		Reference	
Gravidity				
0–1	0.32 (0.17–0.59)	<0.001	0.33 (0.14–1)	0.060
2–4	0.75 (0.44–1.26)	0.280	0.77 (0.37–1.62)	0.501
5+	Reference		Reference	
Information about the effect of undernutrition on pregnancy and child				
Yes	0.7 (0.46–1.01)	0.080	0.77 (0.5–1.19)	0.250
No	Reference		Reference	
Access to food				
Difficult	2.76 (1.8–4.1)	<0.001	**2.78 (1.73–4.47)**	**<0.001**
Not difficult	Reference		Reference	
Difficulty in ANC follow‐up				
Family restriction	0.43 (0.19–0.84)	0.010	0.52 (0.23–1.18)	0.121
Overcrowding CHC	0.83 (0.54–1.28)	0.410	0.88 (0.54–1.43)	0.620
CHC is far away	0.45 (0.16–1.26)	0.130	0.43 (0.13–1.34)	0.140
No problem	Reference		Reference	
Number of meals per day				
Less than three meals per day	4.26 (1.88–9.63)	0.024	2.78 (1.14–6.7)	**0**.**040**
Three meals per day	1.87 (0.97–3.6)	0.058	**1.27 (0.62–2.5)**	**0.510**
More than three meals per day	Reference		Reference	
History of abortion				
Yes	0.7 (0.44–1.08)	0.110	0.84 (0.5–1.43)	0.531
No	Reference		Reference	
Illness during pregnancy				
Yes	0.81 (0.53–1.24)	0.340	0.69 (0.42–1.38)	0.141
No	Reference		Reference	
Using salt‐iodized				
Yes	0.77 (0.52–1.13)	0.190	0.85 (0.52–1.38)	0.520
No	Reference		Reference	
Access to clean water				
Yes	0.76 (0.49–1.17)	0.210	0.78 (0.47–1.3)	0.330
No	Reference		Reference	

*Note:* Significant values are in bold.

Abbreviations: ANC, antenatal care; CHC, community healthcare center; CI, confidence interval.

## 4 Discussion

In this study, we found that 52.0% were undernourished. Multivariable analysis identified significant associations between undernutrition and increasing maternal age, difficulty in accessing food, and consumption of less than three meals per day.

In the present study, the vast majority (52.0%) of the pregnant women had undernutrition during their ANC follow‐up. This prevalence is comparable to the findings of a study conducted in Ethiopia (38.0%) [42]. However, it is substantially higher than reports from other contexts, including Somalia (21.0%) [43] and a systematic review from Africa (20.0%) [44]. More importantly, a recent Pakistani study in Karachi found 26.6% of pregnant women had MUAC <23 cm [34], and another study among poor and very poor households in rural Bangladesh showed the prevalence of undernutrition was 46.7% [32], and a study of Rohingya pregnant women in Bangladesh reported 34.5% with MUAC <23 cm, with 10.1% having MUAC <21 cm [45]. UNICEF reported that the number of severely undernourished pregnant and breastfeeding women in nations impacted by the ongoing food and nutrition crisis rose by 25.0% from 2020 (5.5 million) to 2022 (6.9 million), with over 1 billion adolescent girls and women globally experiencing undernutrition [46]. The variation across studies may be explained by differences in study populations, measurement approaches, and local living conditions. In addition to these, the broader humanitarian and socioeconomic context of Afghanistan may provide relevant context for understanding the high prevalence observed in this study. Following the political transition in 2021, reductions in external funding and changes in the delivery of health and nutrition programs have been reported [47, 48]. These contextual factors may have affected access to health, nutrition, and food‐support services; however, their specific contribution to the prevalence observed in this study was not directly assessed. Similar effects of political instability on nutrition have been reported in other conflict‐affected countries [49, 50].

In this study, we also found that maternal age was associated with undernutrition [51]. Older pregnant women had higher odds of being undernourished, which might reflect differences in reproductive history, family size, birth spacing, or other unmeasured socioeconomic and nutritional factors [52]. In Afghanistan, older women often have larger families, averaging around 6.2 children, and the median birth interval is only 28.4 months [52]. Repeated pregnancies and closely spaced births may be relevant to maternal nutritional status, but this study cannot directly assess cumulative nutritional depletion.

Difficulty in accessing food was significantly associated with undernutrition in this study. Women who reported difficulty in accessing food were more than two and a half times as likely to be undernourished compared to women who reported no difficulty accessing food. These findings are consistent with evidence from Kenya [53] and Southard's study on food insecurity in women [54], as well as another study in South Asia [13], which underscore that limited access to sufficient and diverse foods is associated with maternal nutritional status [55, 56, 57, 58]. Recent evidence from Afghanistan further suggests that women's nutritional vulnerability occurs within a broader context of economic hardship, food‐system disruption, and barriers to accessing health and nutrition services. Reviews of maternal nutrition in Afghanistan have highlighted the intersection of food insecurity with structural and policy‐level factors, including economic instability, restrictions affecting women's access to employment and healthcare, and disruption of humanitarian and nutrition services [15, 26]. These findings provide important contextual support for the association between difficulty accessing food and maternal undernutrition observed in the present study [59, 60, 61, 62]. However, because these structural factors were not directly measured in our study, they should be regarded as contextual explanations rather than causal mechanisms.

Compared with women consuming more than three meals per day, those consuming less than three meals per day had higher odds of undernutrition. An earlier study among Brazilian families showed that regular family meals are a protective factor against undernutrition [63]. Our finding should be interpreted cautiously. Because the study did not directly assess meal quantity, dietary diversity, dietary quality, or intra‐household food allocation, the underlying reasons for this association cannot be determined from the present data. The finding may reflect residual confounding, measurement limitations, or other unmeasured factors and should therefore not be interpreted as evidence that consuming less than three meals per day increases the risk of undernutrition. Further studies incorporating detailed dietary assessment are needed to clarify this association.

The study found that several factors previously reported to be associated with maternal undernutrition, such as maternal education, occupation, family size, physiological condition of the mother, history of abortion, parity, gravidity, regular ANC follow‐up, family planning utilization, birth spacing, information about the effect of undernutrition on pregnancy and child, illness during pregnancy, use of iodized salt, and access to clean water, did not show a statistically significant association with undernutrition in this study. These findings may differ from previous studies because of differences in study populations, measurement approaches, sample characteristics, or the sociopolitical and humanitarian context of Afghanistan [47, 64, 65, 66]. The lack of significant association with ANC, family planning, and birth spacing may also reflect differences in access to or utilization of these services; however, the present cross‐sectional study cannot determine the reasons for these findings [67, 68]. Given the current political climate and the repression of women's rights, these findings highlight the need for integrated maternal health interventions that address both nutritional and health service deficiencies.

Although maternal age, difficulty in accessing food, and consuming less than three meals per day were statistically associated with undernutrition, statistical significance alone does not establish clinical or public‐health importance. The magnitude and precision of the associations, together with the facility‐based design and potential for residual confounding, should be considered when interpreting their implications for maternal nutrition programs.

### 4.1 Strengths and Limitations

This study contributes to the limited evidence on maternal nutritional status in Afghanistan, a setting where few facility‐based and community‐based studies exist. However, several limitations should be acknowledged. First, this was a single‐site, facility‐based cross‐sectional study conducted at Pol‐e‐Khoshk CHC in Kabul. Therefore, the estimated prevalence of undernutrition cannot be generalized to all pregnant women in Kabul or Afghanistan. The study facility was selected because of its role in maternal and child nutrition services; however, women attending this facility may differ systematically from those who do not seek care or who attend other health facilities. Second, the cross‐sectional design limits causal inference, and the observed associations should therefore not be interpreted as causal relationships. Third, self‐reported data on dietary practices and health behaviors may be subject to recall or social desirability bias, potentially affecting the accuracy of the findings. Fourth, the absence of detailed dietary intake data, household income, and cultural dietary restrictions may have limited the scope of explanatory variables. Therefore, residual confounding is likely. Fifth, access to food was assessed using a self‐reported measure rather than a validated multidimensional food‐security instrument; therefore, the variable may not capture all dimensions of household food insecurity and may be subject to reporting bias. Sixth, the MUAC was the sole measure used to define maternal undernutrition. Although MUAC is practical and does not require gestational‐age‐specific adjustment, reliance on a single anthropometric indicator may result in some degree of outcome misclassification, particularly among women whose measurements were close to the 23 cm cutoff. In addition, because MUAC was the sole measure used to define undernutrition and was measured once, some degree of measurement error or outcome misclassification cannot be excluded. Furthermore, cultural dietary restrictions and seasonal variations in food availability were not assessed, which may also have influenced nutritional outcomes.

## 5 Conclusion

In this facility‐based study of pregnant women attending a CHC in Kabul, half were classified as undernourished. Maternal undernutrition was significantly associated with increasing maternal age, difficulty in accessing food, and meal frequency in this study population. These findings should be interpreted as associations rather than causal relationships given the cross‐sectional study design. Addressing food insecurity and improving access to adequate and diverse foods may be important components of maternal nutrition interventions in Afghanistan.

## Author Contributions

**Zainab Ezadi**: conceptualizing and designing, writing the proposal, reviewing the collected data, data analysis, interpreting the results, writing and editing the original draft of manuscript. **Maliqa Mohaqiq**: collecting data, writing the original draft of manuscript. **Robaba Edalat**: collecting data, writing the original draft of manuscript. **Agus Fitriangga**: writing proposal and writing the original draft of manuscript. **Muhammad Haroon Stanikzai**: interpreting the results, writing and editing the original draft of manuscript. **Massoma Jafari**: editing, reviewing, and revision of the manuscript. All authors have read and approved the final manuscript.

## Funding

The authors have nothing to report.

## Conflicts of Interest

The authors declare no conflicts of interest.

## Data Availability

The primary data used to support the findings of this study are available with the corresponding author upon request.

## References

[1] S. B. Zewude , M. H. Beshah , M. A. Ahunie , D. T. Arega , and D. Addisu , “Undernutrition and Associated Factors Among Pregnant Women in Ethiopia. A Systematic Review and Meta‐Analysis,” Frontiers in Nutrition 11 (2024): 1347851. 10.3389/fnut.2024.1347851 PMC11103006 38769991

[2] E. C. Keats , J. K. Das , R. A. Salam , et al., “Effective Interventions to Address Maternal and Child Malnutrition: An Update of the Evidence,” Lancet Child & Adolescent Health 5, no. 5 (2021): 367–384. 10.1016/S2352-4642(20)30274-1 33691083

[3] F. Siddiqui , R. A. Salam , Z. S. Lassi , and J. K. Das , “The Intertwined Relationship Between Malnutrition and Poverty,” Frontiers in Public Health 8 (2020): 453. 10.3389/fpubh.2020.00453 PMC7485412 32984245

[4] UNICEF , Early Prevention, Detection and Treatment of Child Wasting in the Most Vulnerable Countries to the Global Food and Nutrition Crisis: UNICEF's Acceleration Plan 2022–2023 (UNICEF, 2022), https://www.unicef.org/media/127466/file/No%20Time%20to%20Waste%20Acceleration%20Plan%202022‐2023.pdf.

[5] K. Berhe , L. Weldegerima , F. Gebrearegay , et al., “Effect of Under‐Nutrition During Pregnancy on Low Birth Weight in Tigray Regional state, Ethiopia; a Prospective Cohort Study,” BMC Nutrition 7, no. 1 (2021): 72. 10.1186/s40795-021-00475-7 PMC8594154 34782014

[6] L. R. Brink , T. M. Bender , R. Davies , et al., “Optimizing Maternal Nutrition: The Importance of a Tailored Approach,” Current Developments in Nutrition 6, no. 9 (2022): nzac118. 10.1093/cdn/nzac118 PMC9492153 36157850

[7] C. G. Victora , P. Christian , L. P. Vidaletti , G. Gatica‐Domínguez , P. Menon , and R. E. Black , “Revisiting Maternal and Child Undernutrition in Low‐Income and Middle‐Income Countries: Variable Progress Towards an Unfinished Agenda,” Lancet (London, England) 397, no. 10282 (2021): 1388–1399. 10.1016/S0140-6736(21)00394-9 PMC7613170 33691094

[8] NCD Risk Factor Collaboration , “Trends in Adult Body‐Mass Index in 200 Countries From 1975 to 2014: A Pooled Analysis of 1698 Population‐Based Measurement Studies With 19.2 Million Participants,” Lancet 387, no. 10026 (2016): 1377–1396. 10.1016/S0140-6736(16)30054-X PMC7615134 27115820

[9] E. Ayele , G. Gebreayezgi , T. Mariye , D. Bahrey , G. Aregawi , and G. Kidanemariam , “Prevalence of Undernutrition and Associated Factors Among Pregnant Women in a Public General Hospital, Tigray, Northern Ethiopia: A Cross‐Sectional Study Design,” Journal of Nutrition and Metabolism 2020 (2020): 2736536. 10.1155/2020/2736536 PMC7563058 33083055

[10] M. Muze , M. Yesse , S. Kedir , and A. Mustefa , “Prevalence and Associated Factors of Undernutrition Among Pregnant Women Visiting ANC Clinics in Silte Zone, Southern Ethiopia,” BMC Pregnancy and Childbirth 20, no. 1 (2020): 707. 10.1186/s12884-020-03404-x PMC7678074 33213406

[11] A. Talukder , M. Kelly , D. Gray , and H. Sarma , “Prevalence and Trends of Double Burden of Malnutrition at Household‐Level in South and Southeast Asia,” Discover Public Health 21 (2024): 212.

[12] M. Yigezu , A. Oumer , B. Damtew , et al., “The Dual Burden of Malnutrition and Its Associated Factors Among Mother‐Child Pairs at the Household Level in Ethiopia: An Urgent Public Health Issue Demanding Sector‐Wide Collaboration,” PLoS ONE 19, no. 11 (2024): e0307175. 10.1371/journal.pone.0307175 PMC11534222 39495734

[13] H. Torlesse and V. M. Aguayo , “Aiming Higher for Maternal and Child Nutrition in South Asia,” Maternal & Child Nutrition 14, no. S4 (2018): e12739. 10.1111/mcn.12739 PMC6588023 30499249

[14] M. Fayaz , M. U. T. Butt , A. Khaliq , H. Abbasi , and N. P. H. Haneef , “Factors Associated With Malnutrition Among Pregnant Women and Lactating Mothers in AJK, Pakistan,” Pakistan Journal of Public Health 14, no. Special. NI (2024): 150–154.

[15] S. M. Fayez , “Policy and Structural Drivers of Malnutrition in Afghan Women: Evidence and Interventions,” International Journal of Women's Health 18 (2026): 604409. 10.2147/IJWH.S604409 PMC13221999 42221429

[16] UNICEF , Nutrition: Good Nutrition for the Most Vulnerable Children and Mothers (UNICEF, 2026), https://www.unicef.org/afghanistan/nutrition.

[17] Z. Anwary , M. H. Stanikzai , W. M. Wyar , A. W. Wasiq , and K. Farooqi , “Anemia Among Women Who Visit Bost Hospital for Delivery in Helmand Province, Afghanistan,” Anemia 2021, no. 1 (2021): 9358464. 10.1155/2021/9358464 PMC7817225 33520311

[18] UNICEF , Girls Increasingly at Risk of Child Marriage in Afghanistan (UNICEF, 2026), https://www.unicef.org/press‐releases/girls‐increasingly‐risk‐child‐marriage‐afghanistan.

[19] Z. H. Tharwani , P. Kumar , S. K. Shaeen , Z. Islam , M. Y. Essar , and S. Ahmad , “Maternal Mortality in Afghanistan: Challenges, Efforts, and Recommendations,” Clinical Epidemiology and Global Health 15 (2022): 101038.

[20] United Nations , Afghanistan's Healthcare System on Brink of Collapse, as Hunger Hits 95 Per Cent of Families (United Nations, 2026), https://news.un.org/en/story/2021/09/1100652.

[21] FAO , Afghanistan: Humanitarian Assistance Averted a Food Security Catastrophe in the Harsh Winter Months, But Hunger Persists at Unprecedented Levels (FAO, 2026), https://www.fao.org/newsroom/detail/afghanistan‐humanitarian‐assistance‐averted‐a‐food‐security‐catastrophe‐in‐the‐harsh‐winter‐months‐but‐hunger‐persists‐at‐unprecedented‐levels/en.

[22] IPC, Integrated Food Security Phase Classification , IPC Acute Malnutrition Analysis September 2022‐ April 2023 (IPC, 2026), https://www.ipcinfo.org/.

[23] UNICEF , Nourishing Afghanistan: A UN Call to Accelerate Nutrition Action 2025–2030 (UNICEF, 2025), https://www.unicef.org/afghanistan/documents/nourishing‐afghanistan.

[24] J. Daru , J. Zamora , B. M. Fernández‐Félix , et al., “Risk of Maternal Mortality in Women With Severe Anaemia During Pregnancy and Post Partum: A Multilevel Analysis,” Lancet Global health 6, no. 5 (2018): e548–e554. 10.1016/S2214-109X(18)30078-0 29571592

[25] Z. Ezadi , A. S. Nadi , F. Naziri , F. A. Behroz , R. Rahimi , and M. H. Stanikzai , “Risk Factors for Postpartum Hemorrhage in a Tertiary Hospital in Afghanistan,” Discover Public Health 23, no. 1 (2026): 424.

[26] C. Kim , G. F. Mansoor , P. M. Paya , et al., “Review of Policies, Data, and Interventions to Improve Maternal Nutrition in Afghanistan,” Maternal & Child Nutrition 16, no. 4 (2020): e13003. 10.1111/mcn.13003 PMC7507462 32293806

[27] O. Gianatiempo , S. V. Sonzogni , E. A. Fesser , et al., “Intergenerational Transmission of Maternal Care Deficiency and Offspring Development Delay Induced by Perinatal Protein Malnutrition,” Nutritional Neuroscience 23, no. 5 (2020): 387–397. 10.1080/1028415X.2018.1509178 30124115

[28] WHO , Child Malnutrition (WHO, 2020), https://www.who.int/news‐room/fact‐sheets/detail/malnutrition.

[29] G. N. Ekinci and N. Sanlier , “The Relationship Between Nutrition and Depression in the Life Process: A Mini‐Review,” Experimental Gerontology 172 (2023): 112072. 10.1016/j.exger.2022.112072 36565729

[30] F. M. Sedgi , J. Hejazi , R. Derakhshi , et al., “Investigation of the Relationship Between Food Preferences and Depression Symptoms Among Undergraduate Medical Students: A Cross‐Sectional Study,” Frontiers in Nutrition 12 (2025): 1519726. 10.3389/fnut.2025.1519726 PMC11930683 40129668

[31] A. G. Tilahun , D. A. Fufa , and R. D. Taddesse , “Undernutrition and Its Associated Factors Among Pregnant Women at the Public Hospitals of Bench‐Sheko and Kaffa Zone, Southwest Ethiopia,” Heliyon 8, no. 5 (2022): e09380. 10.1016/j.heliyon.2022.e09380 PMC9111893 35592665

[32] M. A. Haque , N. Choudhury , F. D. Farzana , et al., “Determinants of Maternal Low Mid‐Upper Arm Circumference and Its Association With Child Nutritional Status Among Poor and Very Poor Households in Rural Bangladesh,” Maternal & Child Nutrition 17, no. 4 (2021): e13217. 10.1111/mcn.13217 PMC8476420 34018337

[33] S. Ghosh , K. Spielman , M. Kershaw , et al., “Nutrition‐Specific and Nutrition‐Sensitive Factors Associated With Mid‐Upper Arm Circumference as a Measure of Nutritional Status in Pregnant Ethiopian Women: Implications for Programming in the First 1000 Days,” PLoS ONE 14, no. 3 (2019): e0214358. 10.1371/journal.pone.0214358 PMC6435172 30913234

[34] S. Ambreen , N. Yazdani , A. S. Alvi , M. F. Qazi , and Z. Hoodbhoy , “Association of Maternal Nutritional Status and Small for Gestational Age Neonates in Peri‐Urban Communities of Karachi, Pakistan: Findings From the PRISMA Study,” BMC Pregnancy Childbirth 24, no. 1 (2024): 214. 10.1186/s12884-024-06420-3 PMC10958913 38519904

[35] N. Assefa , Y. Berhane , and A. Worku , “Wealth Status, Mid Upper Arm Circumference (MUAC) and Antenatal Care (ANC) Are Determinants for Low Birth Weight in Kersa, Ethiopia,” PLoS ONE 7, no. 6 (2012): e39957. 10.1371/journal.pone.0039957 PMC3386987 22792140

[36] P. Kumar , N. Sareen , S. Agrawal , N. Kathuria , S. Yadav , and V. Sethi , “Screening Maternal Acute Malnutrition Using Adult Mid‐Upper Arm Circumference in Resource‐Poor Settings,” Indian Journal of Community Medicine: Official Publication of Indian Association of Preventive & Social Medicine 43, no. 2 (2018): 132–134. 10.4103/ijcm.IJCM_248_17 PMC5974833 29899619

[37] D. E. Kpewou , E. Poirot , J. Berger , et al., “Maternal Mid‐Upper Arm Circumference During Pregnancy and Linear Growth Among Cambodian Infants During the First Months of Life,” Maternal & Child Nutrition 16, no. S2 (2020): e12951. 10.1111/mcn.12951 PMC7591302 32835455

[38] United Nations Children's Fund (UNICEF) , UNICEF Programming Guidance. Prevention of Malnutrition in Women Before and During Pregnancy and While Breastfeeding (UNICEF, 2022), https://www.unicef.org/documents/programme‐guidance‐maternal‐nutrition.

[39] M. L. Serbesa , M. T. Iffa , and M. Geleto , “Factors Associated With Malnutrition Among Pregnant Women and Lactating Mothers in Miesso Health Center, Ethiopia,” European Journal of Midwifery 3 (2019): 13. 10.18332/ejm/110131 PMC7839159 33537592

[40] A. K. Belew , T. Awoke , K. A. Gelaye , et al., “Determinants of Malnutrition and Its Associated Factors Among Pregnant and Lactating Women Under Armed Conflict Areas in North Gondar Zone, Northwest Ethiopia: A Community‐Based Study,” BMC Nutrition 9, no. 1 (2023): 102. 10.1186/s40795-023-00758-1 PMC10478398 37667397

[41] M. Fekadu , G. Egata , B. Mengestie , H. A. Adem , and A. A. Usso , “Undernutrition and Associated Factors Among Lactating Mothers in Chiro District, Eastern Ethiopia: A Community‐Based Cross‐Sectional Study,” Frontiers in Global Women's Health 5 (2024): 1440606. 10.3389/fgwh.2024.1440606 PMC11440240 39351344

[42] N. Chea , Y. Tegene , A. Astatkie , and M. Spigt , “Prevalence of Undernutrition Among Pregnant Women and Its Differences Across Relevant Subgroups in Rural Ethiopia: A Community‐Based Cross‐Sectional Study,” Journal of Health, Population, and Nutrition 42, no. 1 (2023): 17. 10.1186/s41043-023-00358-6 PMC10007751 36899418

[43] A. Ahmed , A. Mohammed , M. D. Ateye , and S. Abdusamed , “Prevalence of Undernutrition and Associated Factors Among Adolescent Pregnant Women in Dolo‐Ado Town, Somali Region, Ethiopia,” BMC Pregnancy and Childbirth 25, no. 1 (2025): 105. 10.1186/s12884-025-07153-7 PMC11786326 39891118

[44] P. L. Bestman , A. C. Kaminga , and J. Luo , “Prevalence and Determinants of Maternal Malnutrition in Africa: A Systematic Review,” ARC Journal of Gynecology and Obstetrics 5, no. 1 (2017): 1–12.

[45] S. M. Fahim , S. Das , M. G. Rasul , et al., “Nutritional Status and Dietary Diversity of Pregnant and Nonpregnant Reproductive‐Age Rohingya Women,” Food Science & Nutrition 11, no. 9 (2023): 5523–5531. 10.1002/fsn3.3508 PMC10494628 37701197

[46] United Nations Children's Fund (UNICEF) , Undernourished and Overlooked: A Global Nutrition Crisis in Adolescent Girls and Women , UNICEF Child Nutrition Report Series, 2022 (UNICEF, 2023), https://www.unicef.org/reports/undernourished‐overlooked‐nutrition‐crisis.

[47] Z. Ezadi , N. Mohammadi , R. Sarwari , and S. M. Jafari , “Afghan Women and Access to Health Care in the Past 25 Years,” eClinicalMedicine 43 (2022): 101235. 10.1016/j.eclinm.2021.101235 PMC8669297 34917910

[48] Afghanistan Analyst Network , No Food For Hope: Afghanistan's Child Malnutrition Dilemma in 2023 (Afghanistan Analyst Network, 2023), https://www.afghanistan‐analysts.org/en/reports/economy‐development‐environment/no‐food‐for‐hope‐afghanistans‐child‐malnutrition‐dilemma‐in‐2023/.

[49] World Food Program , Acute Food Insecurity Deepens in the Government‐Controlled Areas of Yemen Amid Severe Funding Cuts and Economic Downturn (World Food Program, 2026), https://www.wfp.org/news/acute‐food‐insecurity‐deepens‐government‐controlled‐areas‐yemen‐amid‐severe‐funding‐cuts‐and.

[50] M. Perez Duque , A. Alburhomy , A. Ahmed , et al., “Acute Malnutrition and Food Insecurity in Yemen, 2021: Evidence From a Two‐Stage Cluster Randomised Survey in a Protracted Crisis,” PLoS Global Public Health 5, no. 7 (2025): e0004331. 10.1371/journal.pgph.0004331 PMC12250524 40644489

[51] S. Poudel , T. Dobbins , H. Razee , and B. Akombi‐Inyang , “Adolescent Pregnancy in South Asia: A Pooled Analysis of Demographic and Health Surveys,” International Journal of Environmental Research and Public Health 20, no. 12 (2023): 6099. 10.3390/ijerph20126099 PMC10298335 37372686

[52] Central Statistics Organization (CSO) , Ministry of Public Health (MoPH) , and ICF , Afghanistan Demographic and Health Survey 2015 (Central Statistics Organization, 2017), https://www.rhsupplies.org/uploads/tx_rhscpublications/Afghanistan_‐_2017.pdf.

[53] S. Keino , G. Plasqui , and B. van den Borne , “Household Food Insecurity Access: A Predictor of Overweight and Underweight Among Kenyan Women,” Agriculture & Food Security 3, no. 1 (2014): 2.

[54] E. M. L. Southard and H. Randell , “Food Insecurity and Women's Mental Health in the Chitwan Valley of Nepal,” SSM—Mental Health 6 (2024): 100337. 10.1016/j.ssmmh.2024.100337 PMC11687357 39742114

[55] K. Kota , R. Pongou , and M. H. Chomienne , “Impact of Household Food Insecurity on the Use of Maternal Health Services in the Savanes Region, Togo: A Qualitative Study,” BMC Public Health [Electronic Resource] 25, no. 1 (2025): 2040. 10.1186/s12889-025-23220-2 PMC12128485 40457279

[56] S. Iqbal and I. Ali , “Maternal Food Insecurity in Low‐Income Countries: Revisiting Its Causes and Consequences for Maternal and Neonatal Health,” Journal of Agriculture and Food Research 3 (2021): 100091.

[57] Y. Kang , K. M. Hurley , J. Ruel‐Bergeron , et al., “Household Food Insecurity Is Associated With Low Dietary Diversity Among Pregnant and Lactating Women in Rural Malawi,” Public Health Nutrition 22, no. 4 (2019): 697–705. 10.1017/S1368980018002719 PMC10260502 30378520

[58] A. Azrimaidaliza , R. Resmiati , G. F. Islami , U. U. Fasrini , and A. D. Apriliani , “Is Food Insecurity Higher Among Pregnant Women in Rural Areas of Indonesia?” Gulhane Medical Journal 67, no. 2 (2025): 108–115.

[59] S. Turkmani and S. Currie , “Afghanistan's Fragile Health System Faces Catastrophe Without Immediate International Funding,” Bmj 389 (2025): r759. 10.1136/bmj.r759 40240071

[60] WHO , Eighty Percent of WHO‐Supported Facilities in Afghanistan Risk Shutdown by June (WHO, 2025), https://www.emro.who.int/afg/afghanistan‐news/eighty‐percent‐of‐who‐supported‐facilities‐in‐afghanistan‐risk‐shutdown‐by‐june.html.

[61] Relief web , Amid Aid Cuts, A Renewed U.S. Policy Increases Health Risks for Women and Girls in Conflict Areas (Relief web, 2025), https://reliefweb.int/report/world/amid‐aid‐cuts‐renewed‐us‐policy‐increases‐health‐risks‐women‐and‐girls‐conflict‐areas.

[62] United Nations , US Funding Pause Leaves Millions ‘in Jeopardy’, Insist UN Humanitarians (United Nations, 2025), https://news.un.org/en/story/2025/02/1159746.

[63] G. A. L. Oliveira , G. Buccini , V. S. S. Gonçalves , M. B. Gubert , and N. Toral , “Regular Family Meals Associated With Nutritional Status, Food Consumption, and Sedentary and Eating Behaviors of Brazilian Schoolchildren and Their Caregivers,” Foods no. 23 (2024): 3975. 10.3390/foods13233975 PMC11641401 39683047

[64] United Nations , Afghan Women Three Years After the Taliban Takeover (United Nations, 2024), https://eca.unwomen.org/en/stories/explainer/2024/08/faqs‐afghan‐women‐three‐years‐after‐the‐taliban‐takeover‐0.

[65] Y. Zhu , M. Azami , M. Fazal , et al., “The Association Between Women's Education and Employment and Household Food Security in Afghanistan,” European Journal of Development Research 36 (2024): 841–867.

[66] M. H. Stanikzai , C. N. Le , S.‐A. Isaramalai , et al., “Barriers and Facilitators of Compliance With Iron‐Folic Acid Supplementation Among Pregnant Women in Southern Afghanistan: A Qualitative Study,” Patient Preference and Adherence 20 (2026): 606199. 10.2147/PPA.S606199 PMC13180690 42158645

[67] M. Valente , A. Lamberti‐Castronuovo , F. Bocchini , et al., “Access to Care in Afghanistan After August 2021: A Cross‐Sectional Study Exploring Afghans' Perspectives in 10 Provinces,” Conflict and Health 18, no. 1 (2024): 34. 10.1186/s13031-024-00594-5 PMC11034132 38649938

[68] E. Tawfiq , M. H. Stanikzai , Z. Anwary , et al., “Quality of Antenatal Care Services in Afghanistan: Findings From the National Survey 2022–2023,” BMC Pregnancy and Childbirth 25, no. 1 (2025): 71. 10.1186/s12884-025-07206-x PMC11770956 39871176

